# Proactive Risk Assessments and the Continuity of Business Principles: Perspectives on This Novel, Combined Approach to Develop Guidance for the Permitted Movement of Agricultural Products during a Foot-and-Mouth Disease Outbreak in the United States

**DOI:** 10.3389/fvets.2016.00117

**Published:** 2017-01-03

**Authors:** Timothy J. Goldsmith, Marie Rene Culhane, Fernando Sampedro, Carol J. Cardona

**Affiliations:** ^1^College of Veterinary Medicine, University of Minnesota, St. Paul, MN, USA

**Keywords:** continuity of business, public–private partnership, risk assessments, permitted movements, foot-and-mouth disease, animal disease, outbreak response, proactive risk assessment

## Abstract

Animal diseases such as foot-and-mouth disease (FMD) have the potential to severely impact food animal production systems. Paradoxically, the collateral damage associated with the outbreak response may create a larger threat to the food supply, social stability, and economic viability of rural communities than the disease itself. When FMD occurs in domestic animals, most developed countries will implement strict movement controls in the area surrounding the infected farm(s). Historically, stopping all animal movements has been considered one of the most effective ways to control FMD and stop disease spread. However, stopping all movements in an area comes at a cost, as there are often uninfected herds and flocks within the control area. The inability to harvest uninfected animals and move their products to processing interrupts the food supply chain and has the potential to result in an enormous waste of safe, nutritious animal products, and create animal welfare situations. In addition, these adverse effects may negatively impact agriculture businesses and the related economy. Effective disease control measures and the security of the food supply thus require a balanced approach based on science and practicality. Evaluating the risks associated with the movement of live animals and products before an outbreak happens provides valuable insights for risk management plans. These plans can optimize animal and product movements while preventing disease spread. Food security benefits from emergency response plans that both control the disease and keep our food system functional. Therefore, emergency response plans must aim to minimize the unintended negative consequence to farmers, food processors, rural communities, and ultimately consumers.

Outbreaks happen when the right host population meets the right infectious agent at the right time. When highly contagious animal diseases not currently found in a country or region are introduced into a naïve population, the result can be explosive spread. For this reason, the response to such a foreign animal disease (FAD) must be rapid and well-planned. Historically, response to highly a contagious FAD like foot-and-mouth disease (FMD) has focused on the eradication of the disease and a return to disease-free status as rapidly as possible (1). This approach, commonly referred to as “stamping out,” requires the rapid identification of infected premises, quick depopulation and, for premises with susceptible species in the control area that are at risk for infection but not known to be infected, there are often quarantine and strict movement controls. The scenario in which there are uninfected but susceptible animals near infected premises is likely to occur and is a predicament for regulatory officials. Stopping all movements of animals, products, and potential fomites from infected premises utilizing a stamping out approach is an obvious thing to do to control disease, but what does one do with not known to be infected herds and all of the food they produce?

Everyday there are food products and animals that move from farms in a “just in time” production system. If an FAD outbreak occurs, for those premises in a control area, the product and animal movements can add up quickly. The inability to harvest uninfected animals and move their products to further processing interrupts the food supply chain and has the potential to result in an enormous waste, adversely impact agricultural businesses, and create animal welfare situations. This was more fully recognized following the 2001 outbreak of FMD in the United Kingdom. Of the 6 million animals culled during the 2001 outbreak of FMD in the UK, an estimated one-third died under various types of “welfare cull” (2, 3). These 2 million animals represent the potentially non-infected population that were killed due to the lack of a prepared response that includes continuity of business (COB) considerations for the permitted movement of non-infected animals and animal products. Furthermore, it is increasingly unacceptable globally to destroy large numbers of “healthy” or “non-infected” animals. Thus, current and future response plans should consider COB principles are part of the planning for an FAD response. COB planning is meant to prepare for animal health emergencies and to address what to do with premises and herds that are not known to be infected but may be adversely affected by disease response activities. COB planning tools and guidance can facilitate the managed movement of animals and their products.

Continuity of business principles have been adopted by the United States Department of Agriculture (USDA) Animal Health and Plant Inspection Service (APHIS) Veterinary Services (VS) and were used to guide the permitted movements of products in highly pathogenic avian influenza outbreaks in 2015 and 2016. The stated goals of USDA APHIS VS for a FAD response (4) include COB principles:

The APHIS goals of an FAD response are to (1) detect, control, and contain the disease in animals as quickly as possible; (2) eradicate the disease using strategies that seek to stabilize animal agriculture, the food supply, and the economy and that protect public health and the environment; and (3) provide science- and risk-based approaches and systems to facilitate continuity of business for non-infected animals and non-contaminated animal products.

Achieving these three goals will allow individual livestock facilities, States, Tribes, regions, and industries to resume normal production as quickly as possible. The objective is to allow the United States to regain disease-free status without the response effort causing more disruption and damage than the disease outbreak itself.

Once USDA APHIS VS adopted COB in principle, it became clear that tools were needed to guide the specific decisions that would balance product movement with outbreak control. To that end, a process was developed utilizing risk assessments performed proactively (i.e., before an outbreak happens) to develop and evaluate science-based guidelines and the associated risk of specific movements from premises located in control areas that are not known to be infected. These risk assessments, done before an epidemic occurs, also take into consideration the potential factors or strategies to mitigate risk that may be put in place during an outbreak. This proactive risk assessment process is a transparent and scientifically accepted method to evaluate commodity and disease specific pathways of transmission. Proactive risk assessments specifically identify pathways where risk exists and explore the necessary mitigations for reducing the risks. The results of the process can help determine the disease transmission risk of specific product movement and inform the responsible regulatory officials and industry stakeholders who are designing emergency preparedness and COB plans before an outbreak. Ultimately, the planning process will allow for informed decision-making regarding managed movement and the COB plan implementation during an outbreak.

The approach used to develop the proactive risk assessment utilizes a collaborative process involving state and federal regulatory officials, academia, and members of private industry (public–private partnerships) and follows the general risk analysis framework that is presented in the OIE Terrestrial Animal Health Code (5). While the intention of the OIE framework is to prevent the entry of animal pathogens and evaluating the risk of susceptible animal exposure through the importation of live animals, animal products, and commodities into a country, the framework fits well for the situation of addressing risk of spread through movements within a control area.

Throughout this process, the input from the food animal industry is crucial as it helps to supply data to support the work and decide what potential mitigation strategies can be realistically adopted by the industry in the event of an FAD outbreak. State and federal regulators’ input is also important as they provide the procedures and policies for managed movement as well as the logistics to implement the emergency preparedness plans. The effect of proposed mitigations on existing risk pathways is evaluated and then used to inform the development of movement guidelines that can then become permitted movement guidance. Without development of proactive risk assessments, similar decisions about movements would have to be made quickly during an outbreak and sometimes without a full understanding of risk. This is one of the main advantages of this approach. The collaborative, proactive approach makes it possible to compile the best available information, model scenarios, understand movement risks, and form mitigation strategies relevant to current production practices. The mitigation strategies can then be put in place and the movements can occur all while following the prescribed regulatory structure. This allows COB to be included in preparedness planning and increases the adoption and awareness of guidelines and tools pre-event.

The collaborative public–private partnership approach is thus a key component to the development of the proactive risk assessments. Just as they would need to work together in an outbreak, these sectors collaborate in developing the proactive risk assessments and guidelines for managed product movement for specific commodities. The process itself communicates findings to the collaborating groups and supports the development of networks of individuals that include public and private stakeholders. Regulators learn about food animal industry systems and practice while industry representatives learn about regulatory processes and requirements. This is a key part of the risk communication step of risk analysis.

Ultimately, the decision to allow managed movements in an outbreak from individual premises in an established control area will be the decision of the responsible regulatory official. The COB plans and guidance materials are practical and useable tools for decision makers. However, it is important to recognize that final decisions in an outbreak situation may have many other constraints like resource limitations, political restrictions, or biological considerations that have not been anticipated in the development of the risk assessment. Also, one of the main limitations is that no one can know exactly what the next outbreak will look like. Although guidance documents can incorporate what is known from past outbreaks, biological agents have a way of acquiring novel characteristics and presenting themselves in new ways. For that reason, guidelines developed through this process to support COB are just that—*guidelines*—and not requirements. The judgment needed to balance disease control, and COB must be made in the context of the ongoing outbreak; this is the intense burden of the responsible regulatory officials.

In the end, outbreaks are expensive, time consuming, and a serious threat to food security and business. No one wants them to happen but when they do, the negative impacts on farmers, food processors, rural communities, and consumers can be lessened with planned responses that include the development of proactive COB guidelines. Regulatory requirements that stop the movement of all animals and animal-derived products may likely result in disease eradication but may just as likely have serious deleterious effects on the entire food supply chain. The development of COB plans that simultaneously address the challenges of controlling an FAD outbreak, maintaining the supply of food to the consumer, and ensuring the viability of the food industry represent an important step in FAD response.

## Author Contributions

TG was the lead author of the manuscript and formed the perspectives. FS, CC, and MC contributed to the writing and editing of the manuscript.

## Conflict of Interest Statement

The authors declare that the research was conducted in the absence of any commercial or financial relationships that could be construed as a potential conflict of interest. The reviewer AB and handling Editor declared their shared affiliation, and the handling Editor states that the process nevertheless met the standards of a fair and objective review.
